# AC signal characterization for optimization of a CMOS single-electron pump

**DOI:** 10.1088/1361-6528/aa9e56

**Published:** 2018-02-09

**Authors:** Roy Murray, Justin K Perron, M D Stewart, Neil M Zimmerman

**Affiliations:** 1 National Institute of Standards and Technology, Gaithersburg, MD, United States of America; 2 Department of Physics, California State University San Marcos, San Marcos, CA, United States of America

**Keywords:** quantum dot, charge pumping, silicon, transfer function

## Abstract

Pumping single electrons at a set rate is being widely pursued as an electrical current standard. Semiconductor charge pumps have been pursued in a variety of modes, including single gate ratchet, a variety of 2-gate ratchet pumps, and 2-gate turnstiles. Whether pumping with one or two AC signals, lower error rates can result from better knowledge of the properties of the AC signal at the device. In this work, we operated a CMOS single-electron pump with a 2-gate ratchet style measurement and used the results to characterize and optimize our two AC signals. Fitting this data at various frequencies revealed both a difference in signal path length and attenuation between our two AC lines. Using this data, we corrected for the difference in signal path length and attenuation by applying an offset in both the phase and the amplitude at the signal generator. Operating the device as a turnstile while using the optimized parameters determined from the 2-gate ratchet measurement led to much flatter, more robust charge pumping plateaus. This method was useful in tuning our device up for optimal charge pumping, and may prove useful to the semiconductor quantum dot community to determine signal attenuation and path differences at the device.

## Introduction

Pumping single electrons at a known frequency is being widely pursued for a quantum based current standard [1, 2]. These devices, which convert a driving signal frequency *f* to a current *I* = *nef*, where *e* is the electron charge, and *n* is an integer, will likely become the basis on which the ampere is defined [3]. In order to operate these devices as robust standards, increasing the current and accuracy of a pump is of utmost importance.

To use single-electron pumps as a current standard, the current produced will need to be higher than a single pump is likely to produce. Parallelizing these devices will require stable, robust operation, where setpoint drift is minimized. Many of the platforms currently utilized for pumping have been shown to drift due to changes in the local charge offset [4, 5]. This work utilizes a highly stable, industry compatible CMOS device, shown to not suffer from many of the charge offset drift issues in other platforms [6]. Tuning these stable CMOS devices individually is a necessary first step towards building multi-device pumps.

The desire for an accurate current standard has spurred many recent studies and a variety of pumping methods in semiconductor tunable barrier devices. One of these methods uses a single AC signal with no applied source-drain bias [7–11]. This work has produced recent uncertainties as low as 1.6 × 10^−7^ at 500 MHz (80 pA of current) [11]. Using 2 separate AC signals to pump charges has also been pursued, utilizing many different methods. Some high accuracy, relatively high current results have been achieved using methods other than the ratchet and turnstile which we discuss in this paper, such as single atoms [12] or double quantum dots [13, 14]. However, most studies using two AC signals have focused on the ratchet [15–18] or turnstile [19–21] mode.

In terms of two AC signals, the present state of the art lowest uncertainty is about 2 × 10^−6^ at 1 GHz [22], an order of magnitude higher than the best results using a single AC signal. No study has been published on error rates in the same device using two different modes with both a single AC signal and two AC signals. Thus, the higher error rate in the best two signal experiment (2 × 10^−6^) might be due to the specifics of the measurement setup, operating mode or device non-idealities. However, the difficulty in coordinating two separate high frequency AC signals at the device is a likely limiting factor. In this study we outline a method for characterizing the AC signals at the device, allowing us to measure and correct for accumulated phase and attenuation differences between the two signals, ultimately leading to flatter plateaus at higher frequencies.

Some work has been done on calibrating the amplitude of a square wave measured at the device [23], but this relies on well-shaped square waves, which can suffer from distortion at higher frequencies. Our method uses sine waves, which do not become distorted, and the measurement can be performed over a wide range of frequencies. Another study explored the amplitude and phase difference of two AC signals at a device [24], though they did not quantify their method nor use it to map out a transfer function at their device. In this study, we examine the transfer function and phase difference at a range of frequencies using a CMOS single-electron pump.

**Figure 1:**
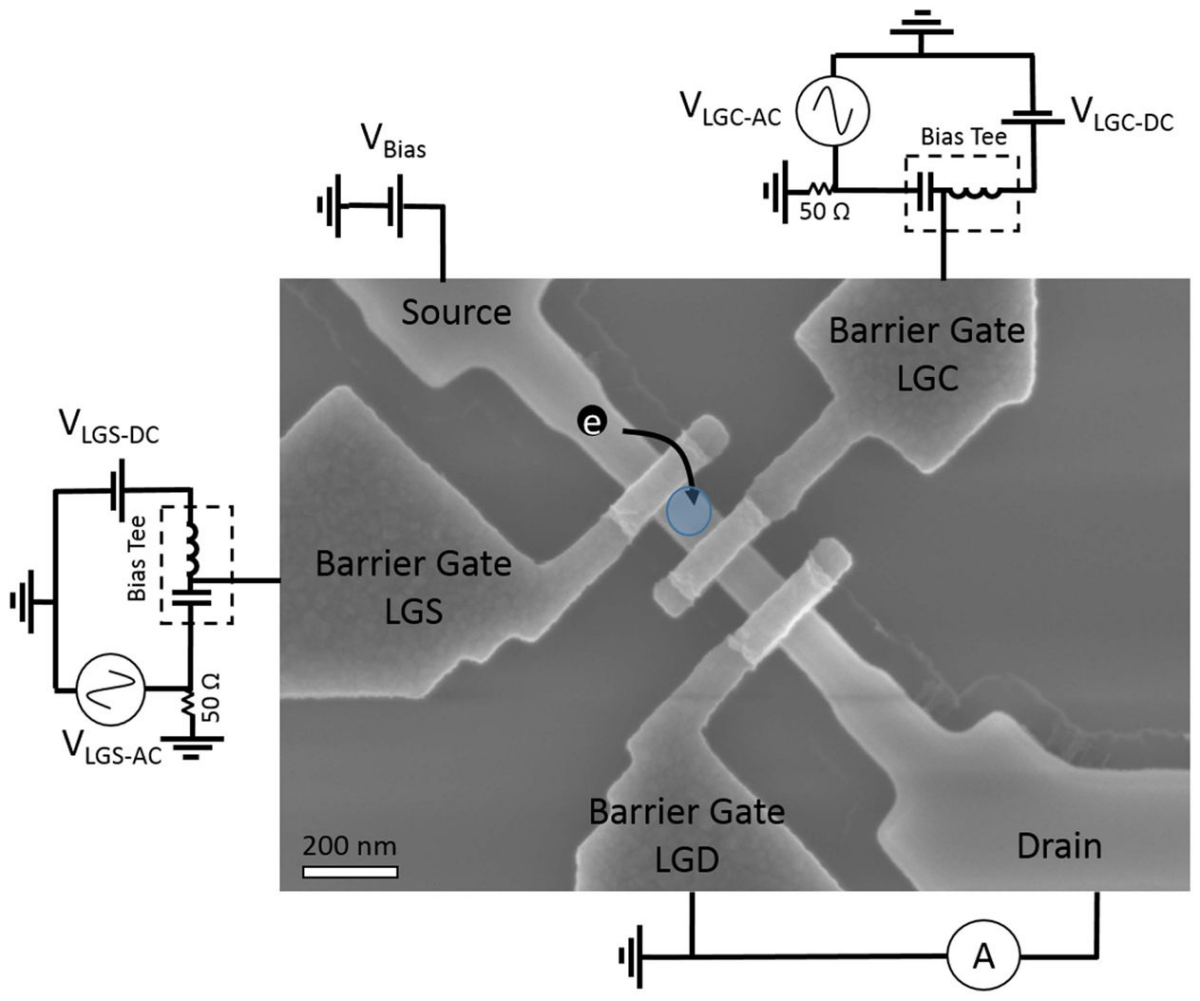
SEM image of a similar device to the one used in this study, where the upper gate, which covers the visible part of the nanowire, is not present. The device used in this study has a 90 nm wide wire and 100 nm long gates. Also pictured is the circuit diagram, where the bias tees and 50 Ω resistors to ground are all located very near the device, in the sample box mounted on the mixing chamber. LGD was grounded due to an unintentional dot located under the gate.

Figure 1 shows an scanning electron microscopy image of our device with the circuit diagram overlaid. Our device consists of a 90 nm wide Si nanowire mesa etched from a silicon-on-insulator wafer. Patterned on top of the nanowire are 3 poly-Si barrier gates, each 100 nm long and spaced by 100 nm. After growing a thin oxide, an upper gate is patterned from poly-Si, covering the Si nanowire. In typical operation, the upper gate is used to invert the Si nanowire, forming a 2D electron gas (2-DEG), while the barrier gates are used to cut off the 2-DEG and form a quantum dot. We fabricated these devices with a fully CMOS process flow developed at the Center for Nanoscale Science and Technology nanofabrication user facility at the National Institute of Standards and Technology. More details of the device fabrication can be found in our previous work [6].

The measurements take place in a cryogen free dilution refrigerator (DR) at a base temperature of 8 mK. Application of the AC signals increases the temperature to 250 mK as measured by a thermometer on the mixing chamber. AC signals are transmitted to the device through co-axial lines and a bias tee at the mixing chamber. No intentional attenuation was added to the AC lines. Just before the bias tee, we have a 50 Ω thin film resistor with axial leads (non-surface mount) to ground to prevent signal reflection and distortion. This 50 Ω resistor was placed in the sample box within *λ*/4 of the device for all frequencies explored in this study. Just after the resistor, a homemade bias tee was used to combine AC and DC signals. The bias tee consisted of a 10 nF ceramic capacitor (measured to be 500 pF at 4 K) and a 470 uH inductor (253 uH at 4 K). Both were discrete non-surface mount components connected with solder on the axial leads. All DC signals travel to the device through co-axial lines and pass through either a low pass filter, a meander line on a printed circuit board at the mixing chamber, or both to lower the electron temperature at the device. AC signals were generated with a Tektronix AWG 70002a 2 channel 25 GSa/s arbitrary waveform generator. Current was measured using a Femto DLPCA-200 current pre-amplifier.

## Pumping modes

All pumping in this paper uses two AC signals applied to gates LGS and LGC. *V*_LGC-AC_ is kept at 250 mV peak amplitude at the generator, while *V*_LGS-AC_ is varied. In the ratchet mode, a small source-drain bias of 3.4 mV is applied to cancel the offset created by the current pre-amplifier, creating an effective bias of less than 0.1 mV. While discussing the pumping modes used in this study, we assume the electrostatic lever arms for each gate are equal. This is roughly true for the device used here, where the difference in lever arms is about 5%.

**Figure 2:**
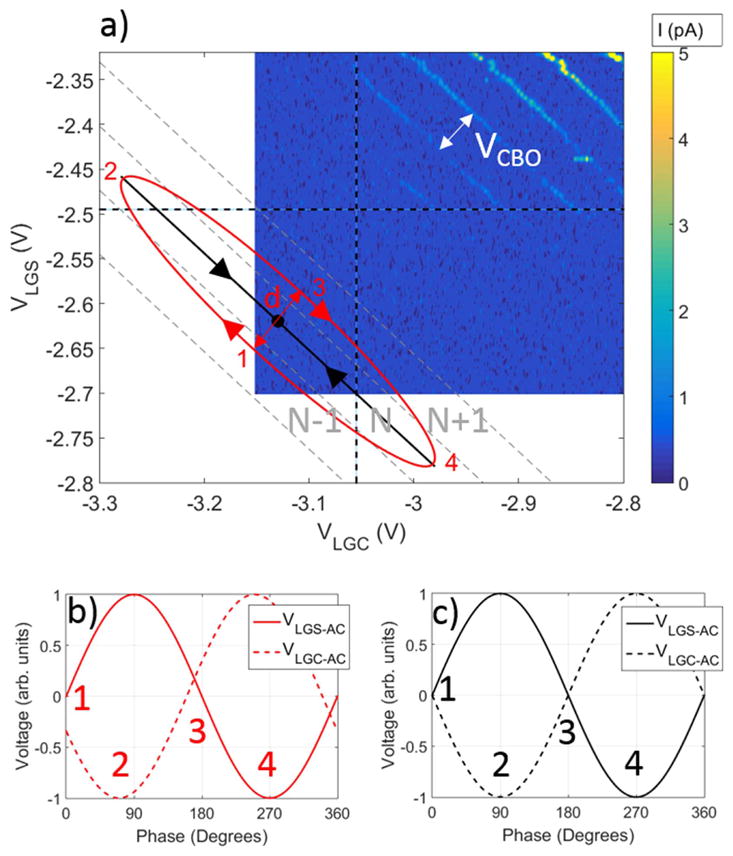
(a) DC 2-gate turnoff curve where *V*_UG_ = 2.3 V and *V*_Bias_ = 0.8 mV above the current pre-amplifier offset, with approximate points of turnoff for each barrier gate labeled with vertical and horizontal dashed black lines and locations of additional non-visible coulomb blockade resonances highlighted with light gray diagonal lines. The black dot (*V*_LGS-DC_ = −2.62 V, *V*_LGC-DC_ = −3.13 V) indicates the DC voltages used for pumping in this study, and the black (red) line indicates the path of the applied AC voltage for turnstile (ratchet) pumping. Data was not taken in the white region inside of the axes to avoid damaging the device with overly negative gate voltages. (b) AC voltages used for ratchet pumping with a 160° phase shift and (c) AC voltages used for turnstile pumping.

The 2-gate ratchet pumping mechanism is illustrated in figures 2(a) and (b). At point 1, the dot’s electronic chemical potential *μ_N_* is unoccupied, leaving *μ_N_*_−1_ as the highest occupied state. Also at point 1, the AC voltage on LGS is at 0, while the AC voltage on LGC is slightly negative (resulting in a higher barrier for electrons). From point 1 to 2, the LGS barrier drops, while the LGC barrier rises. The phase difference between the two signals results in *μ_N_* increasing. Every time the trajectory crosses a sloped dotted white line, *μ_i_* moves above or below the Fermi energy. At point 2, the LGS barrier is low and *μ_N_* is below both the source and the drain reservoir Fermi energy levels. This causes an electron to tunnel through the LGS barrier and onto the dot. From 2 to 3, LGS raises while LGC lowers and the dot level shifts further down, bringing *μ_N_*_+1_ below the source’s Fermi energy. This allows an additional electron to tunnel onto the dot before the ellipse crosses the horizontal dashed black line, representing where the tunnel barrier is too high to permit electron flow. The total shift in *μ* from point 1 to 3 is two electrons, which brings both *μ_N_* and *μ_N_*_+1_ below the Fermi energy. Between points 2 and 3, when the trajectory is above the dashed black line, an electron can still tunnel across LGS. However, once two electrons have been loaded, the dot is in Coulomb blockade and no extra electrons can be loaded. From point 3 to 4, LGS raises while LGC lowers, causing *μ* to rise. The combination of *μ_N_* and *μ_N_*_+1_ being above the source and drain reservoir Fermi levels and the LGC barrier being low causes two electrons to tunnel through the LGC barrier to the drain. Every cycle causes two electrons to be transferred from the source to the drain, resulting in a current of 2*ef*. The number of electrons pumped per cycle can be varied by changing the applied DC voltages or the phase between the two AC signals.

**Figure 3:**
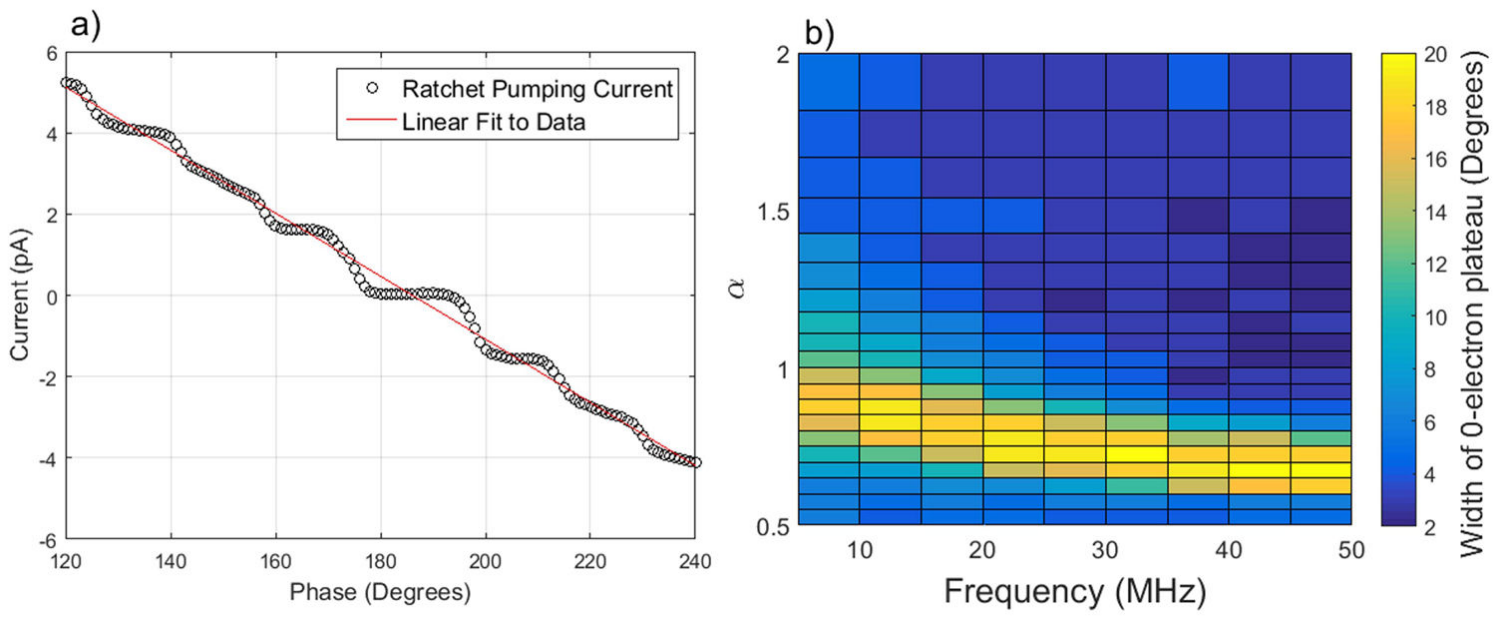
(a) Typical 2-gate ratchet phase sweep taken at the DC voltage indicated in figure 2(a) but with no bias voltage and at 5 MHz with *α* = 0.85. The line is a linear fit to equation (2). (b) Width of the 0-electron plateau as a function of both *α* and *f*, where the 0-electron plateau is defined as all points within *ef*/5 of 0 current.

Current can be controlled in the 2-gate ratchet by varying the phase difference between the two AC signals. Jehl *et al* explored tuning this phase shift in order to achieve electron pumping with various numbers of electrons per cycle [17]. The ellipse seen in figure 2(a) corresponds to pumping two electrons per cycle. However, if the phase difference is increased slightly, the ellipse will narrow, transitioning to pumping zero electrons per cycle. If the DC pumping point is shifted up or down to sit directly on a transition line, the same size ellipse would correspond to one electron pumped per cycle. In a typical 2-gate ratchet sweep, the relative phase between the two gates is varied, creating a plot like the one seen in figure 3(a). A phase offset of 160° traces out the ellipse seen in figure 2(a).

In contrast to 2-gate ratchet pumping, the turnstile requires a source-drain bias. We apply this bias to the source, leaving the drain’s energy level grounded through the current pre-amplifier. At a fixed bias, figure 2(c) illustrates one pumping cycle. At point 1, both barrier gates are equal and opaque to current, shutting off all tunneling. At point 2, LGS has lowered sufficiently to allow one electron to tunnel from the source. LGC has increased, both ensuring that no electrons flow from the dot to the drain at this point, and canceling out the capacitive coupling from LGS, leaving *μ_N_* constant. At point 3, both LGS and LGC are again equal, but now the dot is occupied by an electron. At point 4, LGC lowers and the electron tunnels out onto the drain. After one cycle, one electron has been pumped from the source to the drain, resulting in a current of *ef*. This pumping mode requires both the AC amplitudes of each gate to be equal and the phase difference between the two AC signals to be exactly 180°. Our ratchet scans sweep *I* versus *φ*, releasing the constraint on Δ*φ*(*f*) required by the turnstile. For this reason, we first optimized the ratchet.

Flat plateaus and well determined pumping demand that the two AC signals have well known phase offsets and equal amplitudes at the device with equal capacitive lever arms. The relative phase and amplitude are easily controlled at the signal generator, but due to non-idealities in the transmission lines, they can vary significantly at the device. For the 2-gate ratchet, an *I* versus *φ* sweep can reveal phase offsets, which can then be adjusted for at the source. Unequal AC amplitudes can also negatively affect pumping, and we will discuss more on the optimization of this parameter later.

Optimization of pumping should result in flat plateaus, which will occur at *I* = *nef*. Properly optimizing the device can prove difficult due to the 7-parameter phase space. The 7 degrees of freedom in this experiment are *V*_UG_, *V*_LGS-DC_, *V*_LGC-DC_, *V*_LGS-AC_, *V*_LGC-AC_, *φ*, and *V*_Bias_. For simplicity, *V*_UG_ was kept constant. *V*_LGS-DC_ and *V*_LGC-DC_ were chosen using 2-gate sweeps as described in the next section. We used 2-gate ratchet pump *I* versus *φ* scans at various AC amplitudes to optimize *V*_LGS-AC_ and *V*_LGC-AC_ by maximizing the 0-electron plateau, then determined Δ*φ*(*f*) from those measurements. These optimal parameters were used in the 2-gate turnstile, where we optimized *V*_Bias_.

## 2-Gate ratchet optimization

To determine the optimum pumping parameters for these devices, we started by setting the DC voltages to obtain a well-defined quantum dot between two of the barrier gates. These voltages were chosen from single gate sweeps of the gates and bias voltage. The upper gate voltage was chosen to be 2.3 V by sweeping *V*_UG_ without any intentional barriers and choosing a value where the channel was fully turned on. *V*_UG_ remained fixed for this study. The barrier gate LGD was found to have an unintentional dot located beneath it and was therefore not used in this work. Single gate turn-off sweeps of LGS and LGC showed both gates turned off conduction. We performed a 2-gate sweep as seen in figure 2(a), confirming that an intentional dot was formed between barrier gates LGS and LGC. We did not take data all the way to the axes shown in figure 2(a) because of a chance of breaking down the thin oxide barrier between the upper gate (2.3 V) and the lower gate when too large of voltages occur between the two gates. This sweep also showed that the capacitance of each barrier gate to the dot is approximately equal (*C*_LGS_/*C*_LGC_ = 1.05), resulting in lines near a 45° angle. We then repeated the same sweep with 5 MHz, 180° phase offset, 250 mV sine waves applied to LGS and LGC, respectively. When the device operates as a charge pump, current appears in the otherwise turned off lower left region of figure 2(a), revealing coulomb blockade resonance peaks. The pumping location used here is seen on figure 2(a) as the black dot located below the lower left edge of conduction. It was chosen to be below the lower left edge of conduction when only DC voltages were applied, and directly between two resonance levels revealed by an applied pumping signal. This pumping location was chosen to be near the expected optimum pumping location, but further optimization could occur with a more thorough parameter search. Once we had chosen the DC voltages for pumping, we began to optimize the AC signals.

Since the two barrier gate capacitances to the dot are nearly equal, ideal pumping should occur when the two AC signals have equal amplitudes at the device. All AC transmission lines were characterized before beginning the measurement, from the generator to the sample box. These measurements showed that, to the sample box, the two transfer functions were identical to within 1 dB. However, once the signal reaches the chip, additional attenuation and reflection factors were present. We began by using equal AC amplitudes on both barrier gates as measured at the generator, and assumed they would still be equal at the device. We define the ratio of AC amplitudes at the generator as:

(1) $$\alpha =\frac{V_{\text{LGS-AC}}}{V_{\text{LGC-AC}}}.$$

Using *α* = 1 as a starting point, we observed plateaus at low frequency pumping, but they shrunk rapidly as frequency increased. To find the *α* that would produce equal AC amplitudes at the device, we maximized the ratchet pumping 0-electron plateau width as a function of *α. α* was varied by maintaining *V*_LGC-AC_ at 250 mV and varying *V*_LGS-AC_. Figure 3(b) shows the result of this optimization at lower frequencies, showing a strong dependence of the 0-electron plateau width on *α*. For this study we defined the width of the 0-electron plateau as the region where the current was within ±*ef*/5. The widest plateau will correspond with the optimal value of *α*, occurring when the AC amplitudes on the two barrier gates are equal at the device. We chose to optimize the 0-electron plateau width since it should be more robust and larger than the 1-electron plateau. Figure 3(b) shows the optimal *α* dropping as a function of frequency, then levelling out. This saturated value of 0.6 continues all the way to 500 MHz, the highest frequency explored in this study. Once the ideal values of *α* for each frequency were identified, we extracted further information from the 2-gate ratchet pumping data to optimize pumping.

Using a simple model comparing the width of the ellipse seen in figure 2(a) to the spacing of the coulomb blockade oscillations, we find the current as a function of the AC amplitude and *φ*:

(2) $$\frac{I}{ef}=-2\sqrt{2}H_{g}(f)\frac{V_{\text{AC-Gen-}g}}{V_{\text{CBO}}}\frac{\varphi -\Delta \varphi (f)}{2},$$

where *H_g_*(*f*) is the transfer function, *V*_AC-Gen_ is the AC amplitude put out by the generator, and *V*_CBO_ is the width between coulomb blockade resonances measured along a line perpendicular to the resonance lines (indicated in figure 2(a)). The derivation of this equation can be found in the supplementary information, which is available online at stacks.iop.org/NANO/29/065202/mmedia. When *φ* − Δ*φ*(*f*) is near *π*, equation (2) is approximately linear, allowing us to fit the 2-gate ratchet pumping current to a straight line as seen in figure 3(a). We choose to ignore the pumping plateaus to simplify fitting and apply the fit to regions where plateaus are non-existent. The slope and *I* = 0 intercept of this line give us two valuable pieces of information: *H_g_*(*f*) and Δ*φ*(*f*) at the device.

**Figure 4:**
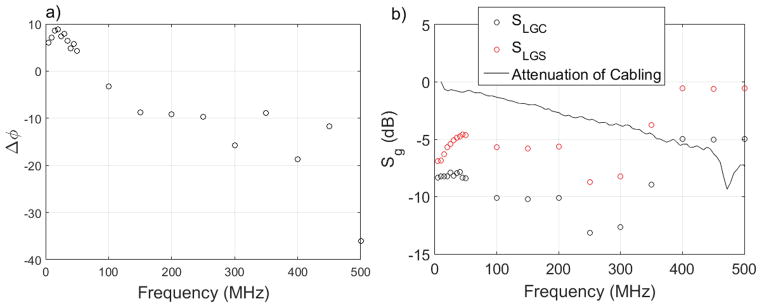
Parameters extracted from equation (2) versus frequency: (a) Δ*φ*(*f*) versus *f*, showing a trend versus frequency due to a difference in cable length, and (b) *S_g_* versus *f* compared to an attenuation measurement of the circuit without the device at 10 mK.

Fitting equation (2) to data similar to that shown in figure 3(a) gives us Δ*φ*(*f*). Plotting out Δ*φ*(*f*) versus *f* reveals the signal path length difference (figure 4(a)). While it is possible to add a length of cable to one of the lines (6 cm to LGC in this case), it is more precise to program the AC generator to the phase offset revealed by the ratchet optimization. Programming the generator is also useful because the data in figure 4(a) do not follow a straight line. This is likely due to resonances and distortion of the signal at the device, which we can analyze better by investigating *H*_g_(*f*).

Fitting equation (2) to the data in figure 3(a) also gives us *H_g_*(*f*). To find the amplitude of the AC signal at the device, we fit a line to the *I* versus *φ* ratchet pumping data taken at an optimized value of *α* (figure 3(a)). Using equation (2), with a measured *V*_CBO_ of 50 mV, the slope of this line reveals the AC amplitude at the device. Figure 4(b) shows the calculated attenuation, defined as:

(3) $$S_{g}=20\log_{10}(H_{g}(f)),$$

where *S_g_* has units of dB and *H_g_*(*f*) is calculated for both LGS and LGC. Also plotted in figure 4(b) is the attenuation measured by a network analyzer at base temperature of the transmission line including the bias tee but without the device. This measurement was made on a bias tee located at the bottom of the DR. Figure 4(b) shows S21 as measured by the network analyzer showing the attenuation of the cable down the DR and the bias tee, with the return cable attenuation (measured separately) subtracted out. This measurement shows that a significant amount of the attenuation on the AC signals occurs at the chip at frequencies up to 300 MHz. The attenuation above 300 MHz is puzzling, and shows some amplification relative to the circuit without the chip. This may be due to some resonances and reflections of the signal at the chip, but is likely also due to some cross capacitances between gates. At high frequencies, these cross capacitances can become significant and have been explored before by Giblin *et al* [25]. Further understanding of the capacitance between gates in this device will likely help to improve pumping, and is planned for future studies.

It should be noted that, while the *H_g_*(*f*) data is valuable, it is not used in the optimization of the device. The optimal values of *α* and Δ*φ*(*f*) obtained from maximizing plateau flatness (figure 3(b)) and fitting equation (2) (seen in figures 3(a) and 4(a)) are used in optimizing pumping. The values of *H_g_*(*f*) are useful in better understanding the mechanics of the device, and will likely prove valuable when using non-sinusoidal pumping signals.

## 2-Gate turnstile

**Figure 5:**
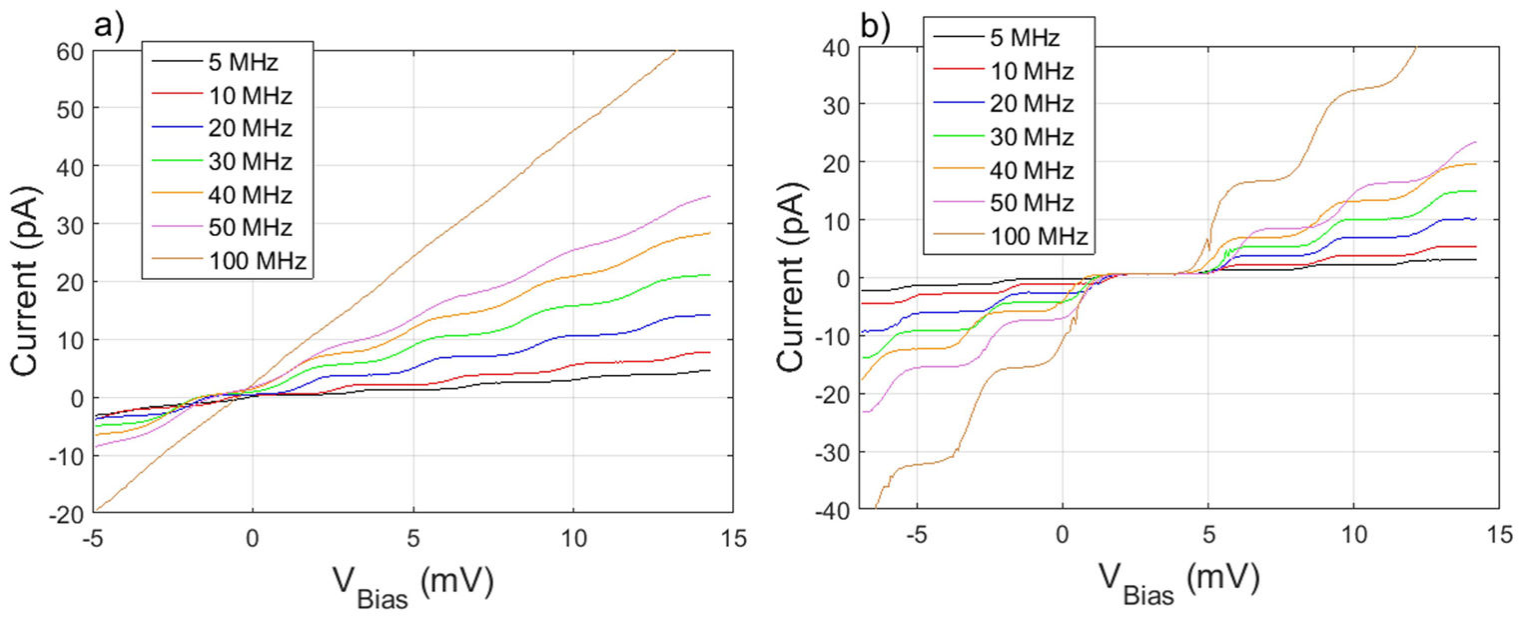
Turnstile pumping scans taken along the black path in figure 2(a) with (a) unoptimized *α* = 1, Δ*φ*(*f*) = 0 AC signals, showing the rapid shrinking of plateaus at higher frequency, and (b) optimized AC signals scanned using the optimal *α* from figure 3(b) and the optimal Δ*φ*(*f*) from figure 4(a).

We proceeded to perform turnstile measurements using the optimized parameters found from the 2-gate ratchet measurements. While low frequency pumping was observed using the assumption that *H_g_*(*f*) = 1 and Δ*φ*(*f*) = 0, at higher frequencies the plateaus disappeared (figure 5(a)). Using the values of *α* and Δ*φ*(*f*) that we found by optimizing the 2-gate ratchet pumping, we significantly increased the plateau width at all frequencies (figure 5(b)). Plateaus remain flat at and above 100 MHz, albeit less wide than the plateaus at lower frequencies.

The plateaus in figure 5(b) also show remarkable stability in the location of the plateaus in bias voltage. In other 2-gate turnstile measurements, the plateau location and the current of the 0-electron plateau seem to drift with frequency [15, 21]. Although the left and right edges of the 1-electron plateau change as a function of frequency, they all share a common bias voltage, allowing the output current to be changed without changing any of the DC voltages.

The 2-gate turnstile results show promising plateaus and require a more thorough optimization of parameters and a reduction in the uncertainty of the current measurement to determine the accuracy of this charge pump. Turnstile plateaus with respect to the source voltage are flat to within 0.1%. Current values are equal to *ef* to within ±1%, limited by the accuracy of the current pre-amplifier. A more accurate measurement setup and more thorough error analysis will be required to reduce the uncertainty on the measurement. 2-gate ratchet measurements had much narrower plateaus than the turnstile, with no plateaus visible above 50 MHz. This may be due to the fact that the AC parameters *α* and Δ*φ*(*f*) were optimized for the 0-electron ratchet plateau with the turnstile in mind. It may also be due to an unintentional dot in series with the pump, requiring some bias voltage to overcome the coulomb blockade of the dot and allow current to flow.

To summarize our optimization of the pumping parameters for a given frequency, listed below is a shortened version of the method listed above:

1. Perform single gate sweeps on *V*_UG_, *V*_LGS-DC_, *V*_LGC-DC_, and *V*_Bias_.
2. Perform 2-gate sweep using LGS and LGC at optimal *V*_UG_, small *V*_Bias_.
3. Repeat 2-gate sweep with 5 MHz sine waves applied to LGS and LGC at a 180° phase offset to pump in the turnstile mode.
4. Choose point below lower left edge of region with DC current found in step 2 and between any regions of pumping current found in step 3.
5. Ratchet style pump of *I* versus *φ* at optimal point chosen in step 4 at various *α* and frequencies. 6. Turnstile pump with optimal *α* and Δ*φ*(*f*) found in step 5.

## Conclusion

In this study, we used 2-gate ratchet pumping to characterize and optimize AC signals at the device. By optimizing the width of the 0-electron plateau found in *I* versus *φ* measurements, we measured the difference in AC amplitudes between the 2 signals at the device. After correcting for this difference by applying different AC amplitudes at the signal generator, we performed 2-gate ratchet pumping at the optimized AC amplitudes. These *I* versus *φ* measurements revealed a change in Δ*φ*(*f*) with frequency, indicating that the path length of the two AC lines was not equal. The slope of the *I* versus *φ* measurements allowed us to determine the AC amplitude at the device, a value difficult to obtain otherwise. While optimization was based on the value of *α* and not on *H_g_*(*f*), *H_g_*(*f*) provides valuable data for analysis of the device and will be very useful in calibrating shaped pumping signals.

Once the AC signals were optimized using the 2-gate ratchet pumping data, we transitioned to pumping electrons with a turnstile. Using the optimized parameters from the ratchet data, the width of all plateaus increased. This suggests that a major limiting factor in 2-gate pumping is coordination of the two AC signals. Our method of 2-gate ratchet parameter optimization corrects for both differences in the transfer function on each signal line and any difference in path length. This leads to higher frequency pumping, resulting in higher current and flatter plateaus. Plateaus can likely be further flattened by optimizing all DC voltages further, but the AC optimization outlined in this study is expected to hold at all DC voltages.

For charge pumps to be used in many different systems with different cabling, high frequency signals need to be calibrated at the device. The method outlined in this study shows a way to characterize these signals using a straightforward, self-calibrating optimization of the pumping signal. This method can also be used for shaped pulses, which may be necessary for pumping at higher frequencies.

## Supplementary Material

Supp1
