# Supplementary material for: AC signal characterization for optimization of a CMOS single-electron pump
Source: Nanotechnology. Author manuscript; Available in PMC 2019 Feb 9. (PMC5963878; doi:10.1088/1361-6528/aa9e56)
Supplement: Supp1 [file NIHMS966673-supplement-Supp1.pdf]

Supplementary Information: AC Signal Characterization for Optimization of a CMOS Single-Electron Pump

Roy Murray[a], Justin K. Perron[b], M.D. Stewart Jr.[a], Neil M. Zimmerman[a]

a) National Institute of Standards and Technology, Gaithersburg, MD USA
b) Department of Physics, California State University San Marcos, San Marcos, CA USA

2-Gate Ratchet Model

To further analyze the AC signals used in the 2-gate ratchet pumping mode, we used a simple model based on the pumping shape traced out in Figure 2a. The elliptical path traced out by the AC signals has a width defined by

$$d = 2\sqrt{2}V_{AC-Dev}\cos\left(\frac{\varphi - \Delta\varphi(f)}{2}\right) \quad \text{Eq. S1}$$

Where *d* is the full width of the ellipse along the diagonal line perpendicular to the coulomb blockade oscillation lines in Figure 2a, $V_{AC\text{-}Dev}$ is the 0 to peak amplitude of both AC signals at the device, φ is the intentional phase difference between the two AC signals, and $\Delta\varphi(f)$ is any unintentional phase shift occurring between the generator and the device.

The current at the device is a simple function of the number of coulomb blockade resonances encompassed by the ellipse seen in Figure 2a. The pumped current can be written as:

$$\frac{I}{ef} = 2\,round\left(\frac{d}{2V_{CBO}}\right) \quad \text{Eq. S2}$$

Where round indicates rounding to the nearest integer and V_{CBO} is the width between coulomb blockade resonances measured along a line perpendicular to the resonance lines (indicated in Figure 2a). This equation assumes a pumping position midway between coulomb blockade resonances, as illustrated by the black dot in Figure 2a, and ignores any non-idealities in the device, such as temperature or other sources of error. Using equation S2 can reveal the experimental width of the ellipse shown in Figure 2a. The width of the ellipse defined in equation S1 takes into account any unintentional phase shift between the two AC signals, but assumes that the two amplitudes are equal. By optimizing α, we can verify that the two amplitudes are identical at the device, but this leads to them having different values at the generator. We define the transfer function on each gate g as:

$$H_g(f) = \frac{V_{AC-Dev}}{V_{AC-Gen-g}} \quad \text{Eq. S3}$$

Where $V_{AC\text{-}Gen\text{-}g}$ is the AC amplitude put out by the generator on gate g. Taking into account any phase shift and attenuation of the AC signals going to the device, and combining equations S1-S3, the current being pumped in the 2-gate ratchet mode is equal to:

$$\frac{I}{ef} = 2\,round\left(\sqrt{2}H_g(f)\frac{V_{AC-Gen-g}}{V_{CBO}}\cos\left(\frac{\varphi - \Delta\varphi(f)}{2}\right)\right) \quad \text{Eq. S4}$$

At low frequencies, this equation fits the data, but at higher frequencies (above 50 MHz) our plateaus shrink and this equation does not fit the data. Ignoring plateaus for simplification of fitting

and application in regions where plateaus are non-existent, and sitting in a region directly between coulomb blockade resonance peaks, we can simplify equation S4 to:

$$\frac{I}{ef} = 2\sqrt{2}H_g(f)\frac{V_{AC-Gen-g}}{V_{CBO}}\frac{\varphi-\Delta\varphi(f)}{2} \qquad \text{Eq. S5}$$

Once we have optimized all DC voltages and α, we use equation S5 to fit all *I* vs φ scans to find $H_g(f)$ and $\Delta\varphi(f)$. These values, taken over a range of frequencies, reveal the transfer function and the signal path length difference at the device. This approximation holds well in the region where φ is near π and breaks down near 0 and 2π. This is due to the ellipse becoming circular as the phase difference increases, and finally becoming an ellipse with its axes pointing perpendicular to the ellipse seen in Figure 2a. For this reason, equation S5 was only used when φ is near π.

Device Optimization

The optimization of pumping parameters required optimizing several different variables. In order to optimize α, scans of *I* vs φ and α were taken. The data seen in Figure 3b was extracted from these plots, where the z axis is a count of the number of data points within *ef*/5 of 0 current. This was used as a proxy for width of the 0-electron plateau at a wide range of frequencies. Figure S1 shows the 0-electron plateau, showing a shift in both α and φ as a function of frequency. It also shows the 0-electron plateau shrinking in width at higher frequencies.

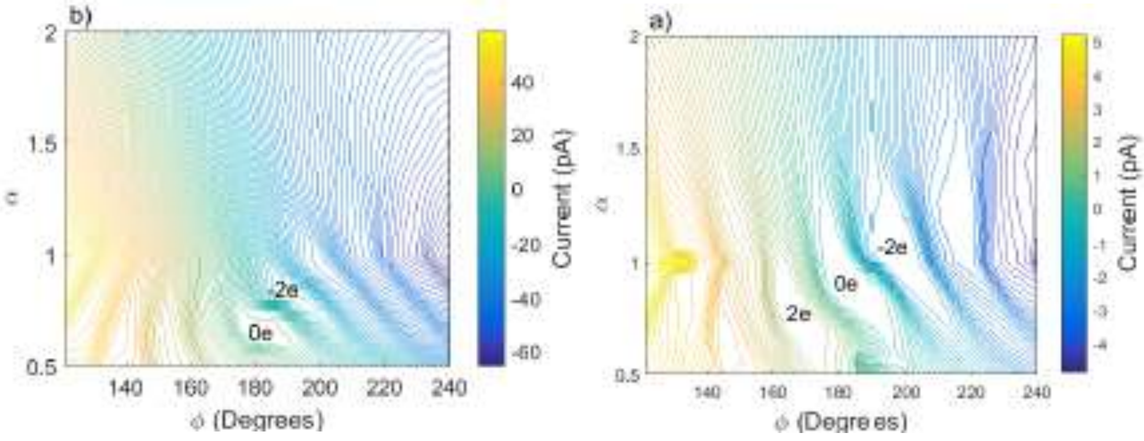

Figure S1: a) 5 MHz with contour lines spaced by 100 fA and b) 50 MHz with contour lines spaced by 1 pA *I* vs φ vs α pumping at V_{Bias} = 0, with plateaus represented by the white regions in the figures
